# Metabolic and bariatric surgery in mild obesity: mid-term outcomes and safety in an Asian population

**DOI:** 10.3389/fendo.2026.1828283

**Published:** 2026-05-20

**Authors:** Wanying Zhong, Lijun Chen, Yuanhao Huang, Dalong Zhu, Wenjuan Tang, Yuqin Ouyang, Xuehui Chu, Wenhuan Feng

**Affiliations:** 1 Department of Endocrinology, Endocrine and Metabolic Disease Medical Center, Nanjing Drum Tower Hospital Clinical College of Nanjing University of Chinese Medicine, Nanjing, China; 2 Branch of National Clinical Research Center for Metabolic Diseases, Nanjing, China; 3 Department of Endocrinology, Endocrine and Metabolic Disease Medical Center, Affiliated Drum Tower Hospital, Medical School, Nanjing University, Nanjing, China; 4 Department of Metabolic and Bariatric Surgery, Affiliated Drum Tower Hospital, Medical School, Nanjing University, Nanjing, China

**Keywords:** Asian population, body mass index normalization, cardiometabolic outcomes, metabolic and bariatric surgery, mid-term outcomes, mild obesity

## Abstract

**Background:**

Metabolic and bariatric surgery (MBS) is an established treatment for severe obesity, but its role in Asian patients with mild obesity (body mass index [BMI] 27.5-32.5 kg/m²) remains controversial due to limited mid-term evidence. This study evaluated the ≥3-year outcomes of MBS in this population compared with patients with BMI ≥32.5 kg/m².

**Objective:**

To evaluate the mid-term (≥3 years) efficacy and safety of MBS in Asian patients with mild obesity, compared with those with BMI ≥MIhe kg/m².

**Methods:**

In this retrospective cohort study, 26 patients with mild obesity and 76 matched patients with higher BMI underwent MBS between 2013 and 2020. Outcomes included percent total weight loss (%TWL), metabolic control, remission of obesity-related comorbidities, and postoperative complications. The primary composite endpoint was defined as glycated hemoglobin <6.5%, low-density lipoprotein cholesterol <2.6 mmol/L, systolic blood pressure <130 mmHg, and homeostasis model assessment of insulin resistance <2.5 mmol/L, μU/mL.

**Results:**

At a mean follow-up of approximately 5 years, the mild obesity group achieved lower %TWL than the higher-BMI group (approximately 20% vs 26%, *P* < 0.05). Despite this, the rate of achieving the composite metabolic endpoint was comparable (30.7% vs 27.6%, *P* = 0.76). Both groups demonstrated sustained improvements in glycemic control, lipid profile, blood pressure, and insulin resistance. Remission of type 2 diabetes, hypertension, hyperuricemia, and metabolic-associated fatty liver disease increased significantly in both groups (all *P* < 0.05). Notably, a substantially greater proportion of patients in the obesity group achieved normalization of body weight (BMI <24 kg/m²). Complication rates were low and comparable.

**Conclusion:**

In Asian patients, MBS appears safe and provides durable metabolic benefits across BMI categories. Despite losing less weight, those with mild obesity achieved similar cardiometabolic improvements and were more likely to reach a normal BMI. These findings are preliminary. Future research should prioritize large, multicenter studies focusing on the BMI 27.5–30 kg/m² subgroup without advanced metabolic disease, randomized trials comparing surgery with current pharmacotherapies, and long-term studies tracking hard cardiovascular endpoints.

## 1 Introduction

Obesity is increasing worldwide. According to the World Obesity Atlas 2025, by 2030 an estimated 1.13 billion adults will have obesity (body mass index [BMI] ≥ 30 kg/m²), representing a sharp global rise since 2010 (1). In Asia, particularly China, this trend is accelerating, with 41% of adults projected to be overweight and 9% obese by 2025 (1–3). Given that China defines obesity at a lower BMI threshold (≥ 28kg/m²), the public health burden is even greater (4). Among affected individuals, those with mild obesity (BMI 27.5–32.5 kg/m²) are the fastest-growing subgroup. This population, often younger and urban, already faces increased risks of type 2 diabetes (T2DM), hypertension, dyslipidemia, cardiovascular disease, and metabolic-associated fatty liver disease (MAFLD) (5, 6). Even so-called “metabolically healthy” obesity often converts to metabolically unhealthy status within a few years (7, 8), underscoring the need for early intervention.

Conventional management of mild obesity relies mainly on lifestyle modification and pharmacotherapy (5), yet outcomes are often modest and difficult to sustain. Although glucagon-like peptide-1 (GLP-1) receptor agonists and other hormone-based agents achieve notable weight loss, they are limited by gastrointestinal effects, and weight regain after discontinuation (9, 10). By contrast, metabolic and bariatric surgery (MBS) produces more profound and durable effects on weight, glycemia, and lipid metabolism, with benefits lasting over 10 years (11, 12).

Historically, MBS was restricted to individuals with BMI ≥ 40 kg/m² or ≥35 kg/m² with severe comorbidities (13). Accumulating randomized evidence demonstrates its superiority over intensive medical therapy for T2DM (11, 12). Consequently, international guidelines now endorse surgery for patients with BMI ≥30 kg/m² (≥27.5 kg/m² in Asians) whose diabetes remains uncontrolled (13–15). The 2022 American Society for Metabolic and Bariatric Surgery/International Federation for the Surgery of Obesity and Metabolic Disorders guidelines further recommend surgery for BMI > 27.5 kg/m² with metabolic disease (13), whereas Chinese consensus statements remain more conservative, suggesting intervention only for BMI ≥32.5 kg/m², or 27.5–32.5 kg/m² with multiple metabolic risk factors (16).

Recent Asian studies report that surgery markedly improves glycemic control and metabolic outcomes in patients with BMI < 35 kg/m² (17–20). Chinese propensity-matched data also show superior 2-year results versus medical therapy in those with BMI < 32.5 kg/m² (18, 21, 22). However, most follow-ups are short, and mid-term evidence on durability and safety remains limited (18, 19, 21, 23).

To address this gap, we conducted a retrospective study comparing ≥3-year outcomes of MBS between patients with BMI 27.5–32.5 kg/m² and ≥32.5 kg/m² at a single high-volume Chinese center. We hypothesized that patients with mild obesity would achieve comparable, durable metabolic improvements without increased surgical risk, providing real-world evidence to inform potential expansion of surgical indications in Asian populations.

## 2 Materials and methods

### 2.1 Study design and patients

This retrospective cohort study was conducted at Nanjing Drum Tower Hospital Clinical College of Nanjing University of Chinese Medicine in China. We reviewed the medical records of patients who underwent laparoscopic MBS—either sleeve gastrectomy (SG) or Roux-en-Y gastric bypass (RYGB)—at the hospital between April 2013 and October 2020.

The eligibility criteria for this study included: (1) age between 18 and 65 years, (2) a preoperative body mass index (BMI) ≥ 27.5 kg/m², and (3) completion of at least 3 years of postoperative follow-up. Exclusion criteria comprised a history of revisional MBS, secondary causes of obesity, severe psychiatric disorders or active substance abuse, and insufficient follow-up data.

Approval for the study protocol was provided by the Institutional Ethics Committee of Nanjing Drum Tower Hospital Clinical College of Nanjing University of Chinese Medicine (Approval No. 2017-030-08). Prior to enrollment, all participants provided written informed consent, and the study was carried out in strict accordance with the ethical principles of the Declaration of Helsinki.

To minimize confounding by baseline characteristics, patients in the higher-BMI group (BMI ≥BMIp kg/m²) were matched to those in the mild obesity group (BMI 27.5-32.5 kg/m²) using a 3:1 nearest-neighbor matching algorithm. Matching was performed based on the following variables: Age: within ±5 years; Sex: exact match; Follow-up duration: within ±6 months. The matching was performed without replacement using a caliper width of 0.2 times the standard deviation of the propensity score (30). A 3:1 matching ratio was chosen to maximize statistical power while maintaining balance between groups, given the smaller size of the mild obesity group (31).

This matching procedure yielded 76 patients in the higher-BMI group and 26 patients in the mild obesity group. As shown in **Table 1**, there were no significant between-group differences in age (*P* = 0.18), sex (*P* = 0.97), or follow-up duration (*P* = 0.07), confirming the success of the matching procedure.

**Table 1:** Characteristics between the two groups at baseline and final follow-up.

Variables	Mild obesity group (*n* = 26)		Higher-BMI group (*n* = 76)		P value	Adjusted mean difference (95% CI)	Adjusted P value
	Baseline	Final follow-up	Baseline	Final follow-up	Baseline		Final follow-up
Male (%)	9 (34.6%)		26 (34.2%)		0.97		
SG (%)	11 (42.3%)		42 (55.3%)		0.25		
Age (years)	41.8 ± 2.2		38.5 ± 1.0		0.18		
Follow-up years	5.0 ± 0.3		4.4 ± 0.2		0.07		
%TWL		18.0 ± 1.7		27.3 ± 1.3		-9.3 (-14.0~ -4.7)	**<0.01**
BMI< 24 kg/m^2^ (%)		11 (43.2%)		7 (9.2%)			**<0.01**
24 kg/m^2^≤ BMI < 28 kg/m^2^ (%)		11 (43.2%)		27 (35.5%)			0.39
Weight (kg)	83.2 ± 1.6	68.2 ± 1.7**	108.5 ± 2.2	78.4 ± 1.6**	**<0.01**	-10.2 (-15.9~ -4.5)	**<0.01**
BMI (kg/m²)	30.4 ± 0.3	24.8 ± 0.5**	39.2 ± 0.7	28.5 ± 0.4**	**<0.01**	-3.8 (-5.3~ -2.3)	**<0.01**
Waist circumference (cm)	101.3 ± 1.5	88.1 ± 1.8**	117.8 ± 1.9	96.7 ± 1.7**	**<0.01**	-8.6 (-14.8~ -2.3)	**<0.01**
Waist-to-height ratio	0.61 ± 0.01	0.53 ± 0.01**	0.71 ± 0.01	0.58 ± 0.01**	**<0.01**	-0.05 (-0.08~ -0.02)	**<0.01**
HbA1c (%)	6.1 ± 0.3	5.9 ± 1.6	6.2 ± 0.1	5.4 ± 0.1**	0.65	0.52 (0.29~0.76)	**<0.01**
FBG (mmol/L)	6.3 ± 0.5	5.3 ± 0.2**	6.2 ± 0.2	4.7 ± 0.1**	0.72	0.56 (0.12~1.0)	**0.01**
2h-PG (mmol/L)	9.6 ± 1.4	7.5 ± 0.6**	9.2 ± 0.5	5.2 ± 0.2**	0.76	2.2 (0.88~3.6)	**<0.01**
F-INS (uU/mL)	15.8 ± 1.4	7.5 ± 0.8**	28.7 ± 2.2	8.6 ± 0.7**	**<0.01**	0.38(-2.1~2.9)	0.76
2h-INS (uU/mL)	83.5 ± 12.0	24.2 ± 7.4**	142.2 ± 11.4	25.1 ± 5.8**	**0.01**	-6.8(-29.6~16.1)	0.56
HOMA-IR (mmol/L, μU/mL)	4.3 ± 0.4	1.7 ± 0.2**	8.3 ± 1.1	1.9 ± 0.3**	0.05	-0.17 (-0.88~0.55)	0.65
SBP (mmHg)	132.2 ± 3.4	121.3 ± 4.6*	137.3 ± 2.2	124.5 ± 2.3**	0.23	-3.2 (-12.6~6.2)	0.50
DBP (mmHg)	82.1 ± 2.5	78.2 ± 3.2	86.3 ± 1.6	80.7 ± 1.5*	0.17	-2.5 (-9.0~4.0)	0.44
TG (mmol/L)	2.0 ± 0.2	1.3 ± 0.2*	1.9 ± 0.1	1.0 ± 0.1**	0.70	0.30 (-0.08~0.69)	0.12
TC (mmol/L)	4.6 ± 0.1	4.3 ± 0.3	4.6 ± 0.1	4.5 ± 0.1	0.61	-0.19 (-0.80~0.42)	0.53
HDL-C (mmol/L)	1.1 ± 0.1	1.6 ± 0.1**	1.0 ± 0.0	1.5 ± 0.1**	0.30	0.13 (-0.08~0.34)	0.21
LDL-C (mmol/L)	2.5 ± 0.1	2.4 ± 0.2	2.8 ± 0.1	2.5 ± 0.1**	0.08	-0.0 (-0.38~0.36)	0.96
ALT (U/L)	32.8 ± 4.7	21.1 ± 2.7*	50.5 ± 4.4	20.2 ± 1.3**	**0.01**	0.46(-5.0~5.9)	0.87
AST (U/L)	22.5 ± 1.9	20.8 ± 1.8	29.8 ± 2.0	19.9 ± 0.9**	**0.01**	0.93(-2.7~4.6)	0.62
γ-GT (U/L)	31.1 ± 3.2	17.2 ± 1.1**	46.6 ± 3.9	20.4 ± 1.7**	**<0.01**	-3.2(-7.2~0.88)	0.12
UA (umol/L)	345.3 ± 16.2	309.2 ± 19.2	404.8 ± 10.1	343.0 ± 9.9**	**<0.01**	-1.0(-36.9~34.0)	0.96
CR (umol/L)	54.0 ± 2.3	83.7 ± 18.7	54.1 ± 1.5	56.7 ± 1.5**	0.97	26.9 (-11.6~66.5)	0.16
Hb (g/L)	139.3 ± 2.9	125.3 ± 4.5**	140.8 ± 1.5	128.2 ± 2.2**	0.62	-2.9 (-12.0~6.2)	0.53
Albumin (g/L)	42.1 ± 0.6	43.1 ± 0.4	42.7 ± 0.5	42.4 ± 0.3	0.58	0.73 (-0.37~1.8)	0.19
Ferritin (umol/L)	19.5 ± 1.3	18.3 ± 1.3	18.1 ± 0.7	18.6 ± 0.6	0.36	-0.24 (-2.8~2.3)	0.85
25-(OH)-D (ng/L)	17.6 ± 1.4	19.2 ± 1.4	15.1 ± 0.7	18.6 ± 0.9**	0.08	0.62 (-2.9~4.1)	0.73
Folate (pg/mL)	14.4 ± 1.3	15.9 ± 1.1	14.6 ± 2.1	14.7 ± 0.7	0.95	1.3 (-1.4~4.0)	0.35
VB12 (pg/mL)	609.3 ± 57.7	499.8 ± 39.9	504.2 ± 20.5	503.2 ± 31.5	**0.03**	-3.5 (-118.6~111.6)	0.95

Values are presented as mean ± SE, n (%), or adjusted mean difference (95% CI). ANCOVA was used to compare continuous outcomes at final follow-up between groups, with the baseline value of each respective parameter entered as a covariate. The adjusted mean difference (mild obesity group minus higher-BMI group) and its 95% confidence interval are reported. *P* values marked in bold indicate statistical significance (*P* < 0.05).

*P < 0.05, **P < 0.01 vs. baseline within the same group (paired t-test).

ALT, alanine aminotransferase; AST, aspartate aminotransferase; BMI, body mass index; CI, confidence interval; DBP, diastolic blood pressure; FBG, fasting blood glucose; F-INS, fasting insulin; HbA1c, glycated hemoglobin; HDL-C, high-density lipoprotein cholesterol; HOMA-IR, homeostatic model assessment for insulin resistance; LDL-C, low-density lipoprotein cholesterol; RYGB, Roux-en-Y gastric bypass; SBP, systolic blood pressure; SG, sleeve gastrectomy; TG, triglyceride; TWL, total weight loss; UA, uric acid; VB12, vitamin B12; 2h-INS, 2-hour insulin; 2h-PG, 2-hour plasma glucose; γ-GT, γ-glutamyl transpeptidase.

### 2.2 Surgical procedure and follow-up

All procedures were performed laparoscopically by an experienced MBS team, following standardized protocols for SG and RYGB (13). A routine preoperative evaluation was conducted to exclude secondary causes of obesity and to identify contraindications to surgery, including uncontrolled psychiatric disorders.

The choice between SG and RYGB was made jointly by the surgeon and patient after informed discussion, based on established clinical guidelines and individual patient characteristics (32). The following factors guided the procedure selection: (1) Gastroesophageal reflux disease (GERD): RYGB was preferred for patients with preoperative GERD symptoms or endoscopic evidence of reflux, as SG may exacerbate reflux. Conversely, SG was favored for patients without GERD due to its technical simplicity and shorter operative time. (2) T2DM severity: RYGB was generally preferred for patients with longer diabetes duration (>5 years), higher baseline HbA1c (>8.0%), or those requiring insulin therapy, given the superior and more rapid glycemic improvement associated with RYGB (32). SG was preferred for patients with mild or early-stage diabetes. (3) Patient preference and anatomical considerations: After discussing the risks and benefits of both procedures, patient preference was respected. Anatomical factors (e.g., prior abdominal surgery, hiatal hernia) were also considered on a case-by-case basis.

After surgery, patients were managed within a structured multidisciplinary program involving metabolic and bariatric surgeons, endocrinologists, dietitians, rehabilitation physicians, and specialized metabolic and bariatric nurses. Follow-up visits were scheduled for one, three, six and twelve months after surgery, followed by annual visits. During each visit, patients received individualized dietary and physical activity counseling, assessment of adherence to vitamin and mineral supplementation. Vital signs and anthropometric parameters were measured, and standard laboratory investigations were conducted. This multidisciplinary follow-up model is consistent with current perioperative care recommendations in MBS and aims to maximize weight loss, ensure nutritional adequacy, and enable early detection and management of complications (13, 33).

### 2.3 Data collection

Both baseline (preoperative) and follow-up data were obtained from electronic medical records and the institutional MBS database. Anthropometric measurements included weight, height, waist circumference, BMI, waist-to-height ratio, and blood pressure.

Venous blood samples were taken after a 10-hour overnight fast for fasting plasma glucose (FPG), glycated hemoglobin (HbA1c), fasting insulin (F-INS), lipid profile [total cholesterol, low-density lipoprotein cholesterol (LDL-C), high-density lipoprotein cholesterol (HDL-C), triglycerides], liver function tests [alanine aminotransferase (ALT), aspartate aminotransferase (AST), γ-glutamyl transferase (γ-GT)], serum uric acid (UA), serum creatinine, and hemoglobin, along with nutritional biomarkers including serum albumin, 25-hydroxyvitamin D, folic acid, and vitamin B12. In addition, 2-hour postprandial glucose (2h-PG) and 2-hour insulin (2h-INS) levels were measured following a standard meal.

In order to assess insulin resistance, a standardized mixed-meal tolerance evaluation was carried out at baseline and every year after that. Plasma glucose and insulin levels were assessed at 0 and 120 minutes. The following formula was used to determine the homeostasis model assessment of insulin resistance (HOMA-IR): HOMA-IR = [FPG (mmol/L) × F-INS (μU/mL)]/22.5 (34).

### 2.4 Outcomes and definitions

Primary outcome. The primary endpoint was a composite metabolic “success” at final follow-up (o 3 years postoperatively), defined as simultaneously meeting all four of the following criteria: HbA1c < 6.5%, LDL-C < 2.6 mmol/L, systolic blood pressure (SBP) < 130 mmHg, and HOMA-IR < 2.5 (mmol/L·μU/mL) (13, 35).

Other outcomes. Other outcomes included weight-related measures (%TWL, BMI, waist circumference), individual metabolic targets (HbA1c, LDL-C, SBP, HOMA-IR), and remission of obesity-related comorbidities (T2DM, hypertension, dyslipidemia, hyperuricemia, and MAFLD).

Diagnostic criteria and remission definitions for obesity-related comorbidities. The following definitions were used for baseline diagnosis and postoperative remission.

T2DM. Diagnosed according to American Diabetes Association and Chinese Diabetes Society criteria: FPG ≥ 7.0 mmol/L, 2-hour oral glucose tolerance test glucose ≥ 11.1 mmol/L, HbA1c ≥ 6.5%, or current use of glucose-lowering agents. Remission was defined as HbA1c < 6.5% for at least 3 months without anti-diabetic medication (24, 25).

Hypertension. Diagnosed as SBP ≥ 140 mmHg and/or DBP ≥ 90 mmHg, or use of antihypertensive medication. Remission was defined as blood pressure < 120/80 mmHg without antihypertensive medication (26).

Dyslipidemia. Defined as any of the following: LDL-C ≥ 3.4 mmol/L, HDL-C < 1.04 mmol/L, triglycerides ≥ 1.7 mmol/L, total cholesterol ≥ 5.2 mmol/L, or current lipid-lowering treatment. Remission required all lipid parameters to be within normal range without lipid-lowering medication (27).

Hyperuricemia. Defined as serum UA ≥ 420 μmol/L (7.0 mg/dL) or use of urate-lowering medication. Remission was defined as serum UA < 360 μmol/L without urate-lowering therapy (28).

MAFLD. Diagnosed according to the Chinese MAFLD consensus: evidence of hepatic steatosis on imaging plus at least one metabolic risk factor (overweight/obesity, glucose metabolism disorder, or multiple metabolic abnormalities). Resolution was defined as absence of fatty liver on follow-up imaging after excluding secondary causes of liver disease (29).

### 2.5 Postoperative complications

Postoperative adverse events were classified as either surgical or nutritional complications. Surgical complications included wound infection, postoperative hemorrhage, anastomotic or staple-line leak, gallstone formation, pneumonia, and dumping syndrome. Nutritional complications included protein–calorie malnutrition (e.g. hypoalbuminemia), iron deficiency anemia (or low ferritin without overt anemia), and bone demineralization (osteopenia or osteoporosis on follow-up imaging). All complications occurring from the index operation to the final follow-up were systematically recorded, and their clinical management was documented in accordance with contemporary MBS standards (13).

### 2.6 Statistical analysis

Statistical analyses were performed using SPSS version 26.0 (IBM Corp., Armonk, NY, USA). Continuous variables are presented as mean ± standard error (SE). Paired t-tests were used for within-group comparisons, and independent Student’s t-tests for between-group comparisons. Categorical variables were analyzed using frequencies and percentages, with chi-square tests for group comparisons (McNemar’s test for paired binary data).

To adjust for baseline differences, Analysis of Covariance (ANCOVA) was performed for all continuous outcomes measured at final follow-up, including %TWL, HbA1c, FBG, 2h-PG, HOMA-IR, SBP, TG, LDL-C, ALT, AST, γ-GT, and UA. For each outcome, the baseline value was entered as a covariate. Adjusted mean differences with 95% confidence intervals (CI) are reported in **Table 1**. For the binary composite metabolic endpoint, multivariable logistic regression was used, adjusting for baseline HbA1c, LDL-C, SBP, and HOMA-IR. A two-tailed *P* < 0.05 was considered statistically significant.

## 3 Results

### 3.1 Baseline characteristics

**Figure 1:**
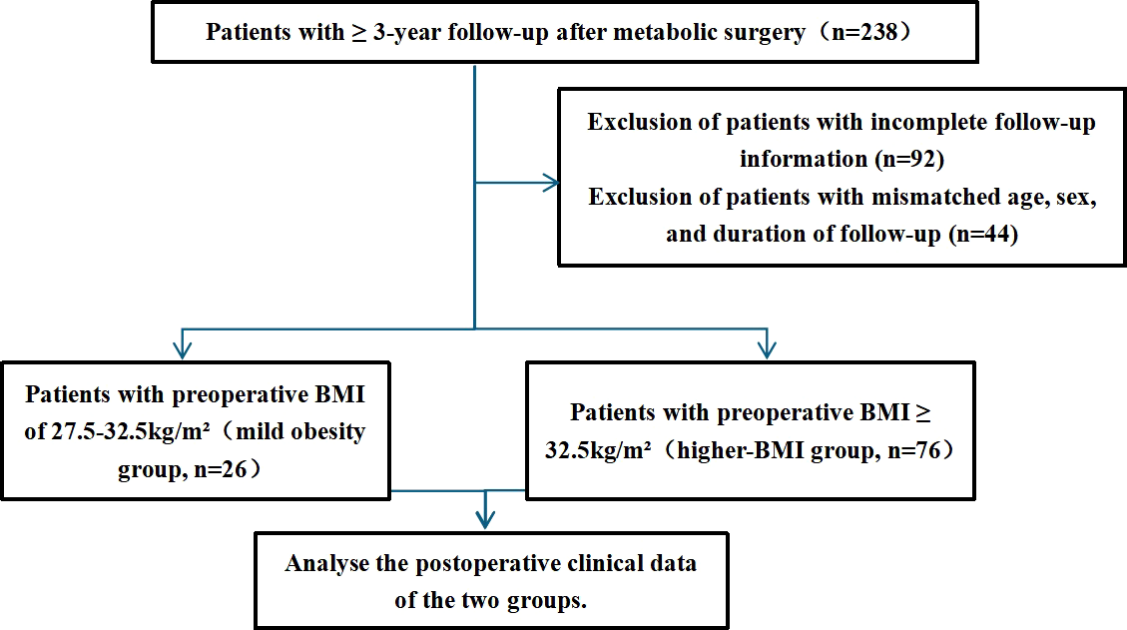
Flow chart of the study.

A total of 102 patients were included and stratified into two groups: the mild obesity group (BMI 27.5–32.5 kg/m²; n=26) and the higher-BMI group (BMI ≥ 32.5 kg/m²; n=76) (**Figure 1**). The two groups were comparable in age, sex, and follow-up duration. The mean age was 41.8 ± 2.2 years with 9 males in the mild obesity group, and 38.5 ± 1.0 years with 26 males in the higher-BMI group. The distribution of surgical procedures (SG vs. RYGB) and the mean follow-up duration (approximately 5.0 vs. 4.4 years, respectively) were also similar between groups (**Table 1**).

Due to the limited sample size, formal subgroup analysis by procedure type was not feasible. However, the distribution of SG and RYGB was similar between the two groups (**Table 1**; *P* = 0.25), and exploratory analyses did not detect a significant interaction between procedure type and BMI group for the primary or other outcomes (data not shown).

**Figure 2:**
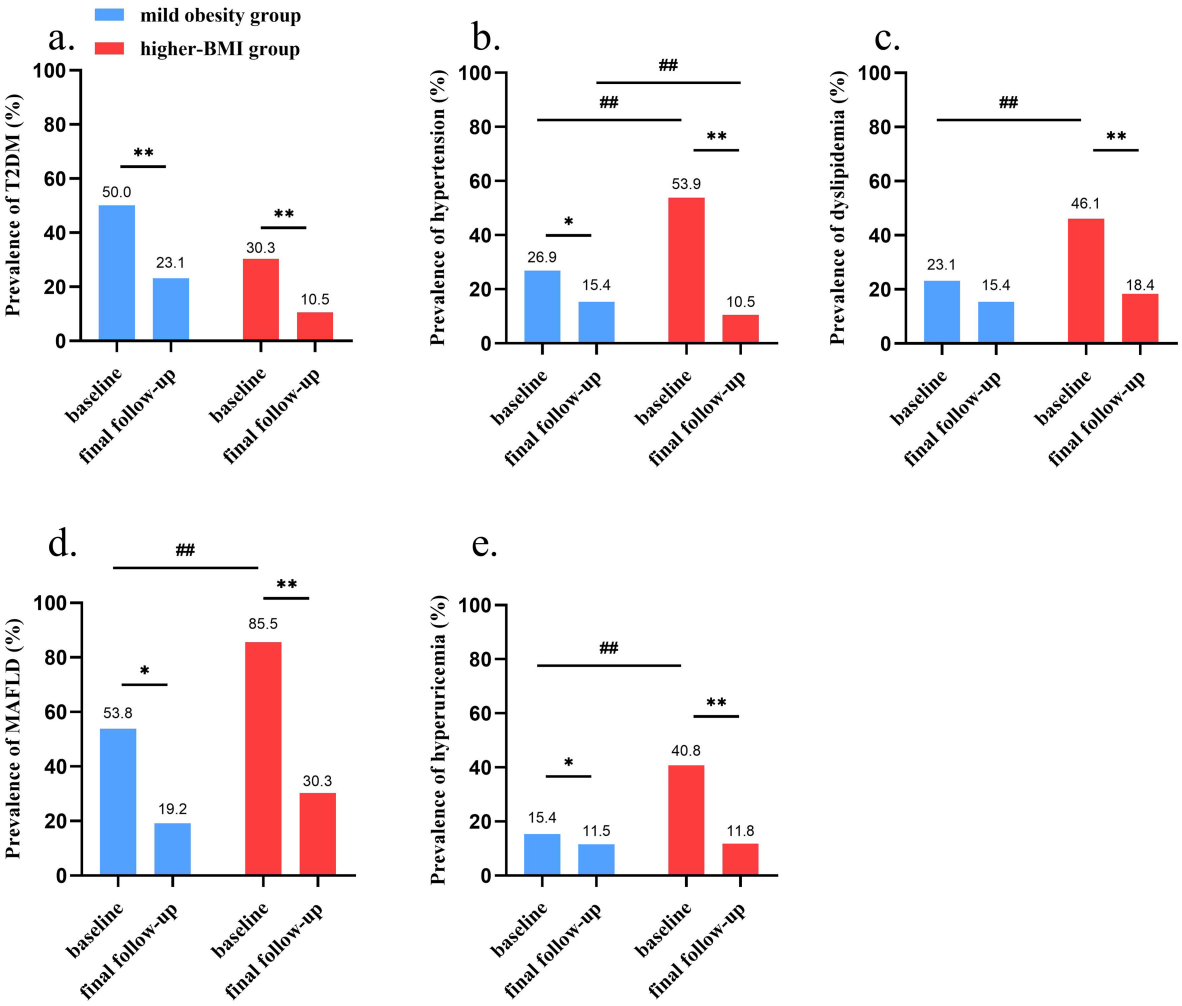
Comparison of obesity-related comorbidities between groups. **(a)** Reduction in T2DM prevalence; **(b)** Reduction in hypertension prevalence; **(c)** Reduction in dyslipidemia prevalence; **(d)** Reduction in MAFLD prevalence; **(e)** Reduction in hyperuricemia prevalence. BMI, body mass index; MAFLD, metabolic dysfunction-associated fatty liver disease; T2DM, type 2 diabetes mellitus. *P < 0.05, **P < 0.01 vs. baseline within the same group; ^#^*P* < 0.05, ^##^*P* < 0.01 vs. mild obesity group at baseline.

At baseline, the higher-BMI group exhibited significantly higher weight, BMI, waist circumference, waist-to-height ratio, F-INS, 2h-INS, ALT, AST, γ-GT, and UA levels (all *P* < 0.05, **Table 1**). Regarding comorbidities, the prevalence of T2DM was comparable between groups, whereas hypertension, dyslipidemia, MAFLD, and hyperuricemia were all more frequent in the higher-BMI group (all *P* < 0.05; **Figures 2a–e**).

### 3.2 Primary outcomes

**Figure 3:**
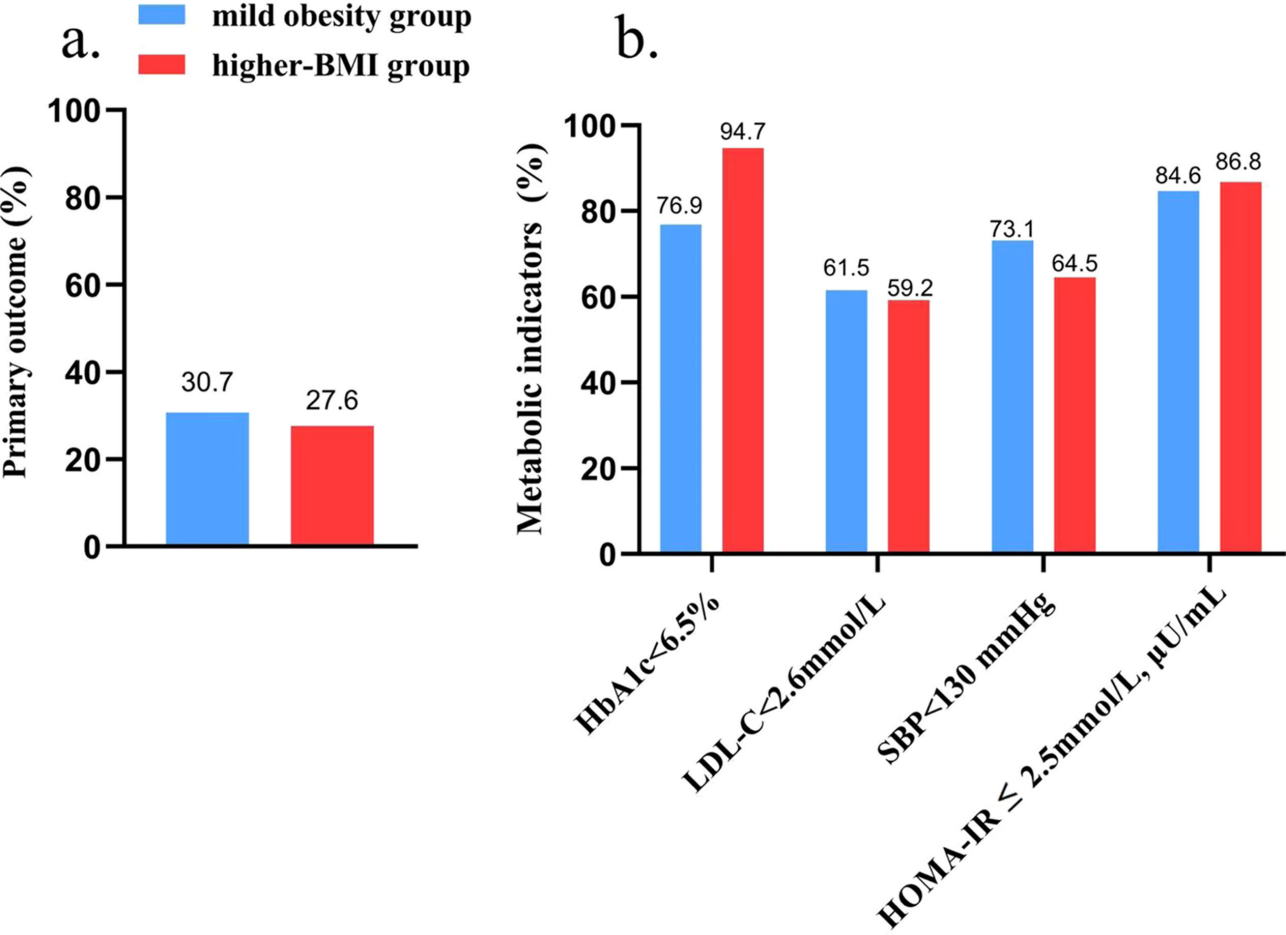
Comparison of primary and individual metabolic outcomes between groups. **(a)** Primary composite outcome: proportion of patients achieving all four metabolic targets simultaneously (HbA1c < 6.5%, LDL-C < 2.6 mmol/L, SBP < 130 mmHg, and HOMA-IR < 2.5); **(b)** Individual metabolic targets: proportion of patients achieving each target separately (HbA1c, LDL-C, SBP, HOMA-IR). HbA1c, hemoglobin A1c; LDL-C, low-density lipoprotein cholesterol; SBP, systolic blood pressure; HOMA-IR, homeostatic model assessment for insulin resistance *P < 0.05, **P < 0.01 vs. baseline within the same group; ^#^*P* < 0.05, ^##^*P* < 0.01 vs. mild obesity group at final follow-up.

At the final follow-up (o 3 years postoperatively), the proportion of patients achieving all four components of the composite metabolic success endpoint was 30.7% in the mild obesity group and 27.6% in the higher-BMI group, with no statistically significant difference between the groups (*P* = 0.76; **Figure 3a**).

### 3.3 Other outcomes

#### 3.3.1 Metabolic indicators

By the final follow-up, there were no significant differences in the proportions of patients achieving target levels for HbA1c (< 6.5%), LDL-C (< 2.6 mmol/L), SBP (< 130 mmHg) or HOMA-IR (< 2.5 mmol/L, μU/mL) between groups (**Figure 3b**).

Both groups showed significant improvements from baseline in glycemic control and insulin resistance, as reflected by reductions in FBG, 2h-PG, F-INS, 2h-INS, and HOMA-IR (all *P* < 0.05, **Table 1**). After adjustment for baseline values using ANCOVA, the higher-BMI group demonstrated significantly better glycemic control than the mild obesity group (HbA1c adjusted difference: 0.52%; 95% CI: 0.29 to 0.76%; *P* < 0.001). In contrast, no significant between-group differences were observed after adjustment for HOMA-IR (*P* = 0.65), SBP (*P* = 0.50), ALT (*P* = 0.87), AST (*P* = 0.62), γ-GT (*P* = 0.12), UA (*P* = 0.96), TG (*P* = 0.12), or LDL-C (*P* = 0.96) (**Table 1**).

Similar improvements were observed in blood pressure and lipid profiles. Both groups demonstrated significant reductions in SBP and TG, along with an increase in HDL-C levels (*P* < 0.01 to 0.05). However, a significant decrease in LDL-C was observed only in the higher-BMI group (*P* < 0.01), despite the absence of between-group differences in LDL-C target achievement (**Table 1**). Overall, both groups achieved clinically meaningful improvements in their lipid profiles.

Regarding liver function and UA, both groups showed significant reductions in ALT and γ-GT levels at the final follow-up (*P* < 0.01 ~ 0.05). In contrast, significant decreases in AST and UA were observed only in the higher-BMI group (*P* < 0.01) (**Table 1**).

#### 3.3.2 Remission of comorbidities

Significant reductions were observed in the prevalence of major obesity-related comorbidities, including T2DM, hypertension, MAFLD, and hyperuricemia in both groups (*P* < 0.01 to 0.05 vs baseline), and the remission rate of hypertension was higher in the higher-BMI group than in the mild obesity group (*P* < 0.01) (**Figures 2a, b, d, e**). Dyslipidemia prevalence also declined, although statistical significance was reached only in the higher-BMI group (46.1% vs 18.4%, *P* < 0.01, **Figure 2c**).

#### 3.3.3 Weight loss

**Figure 4:**
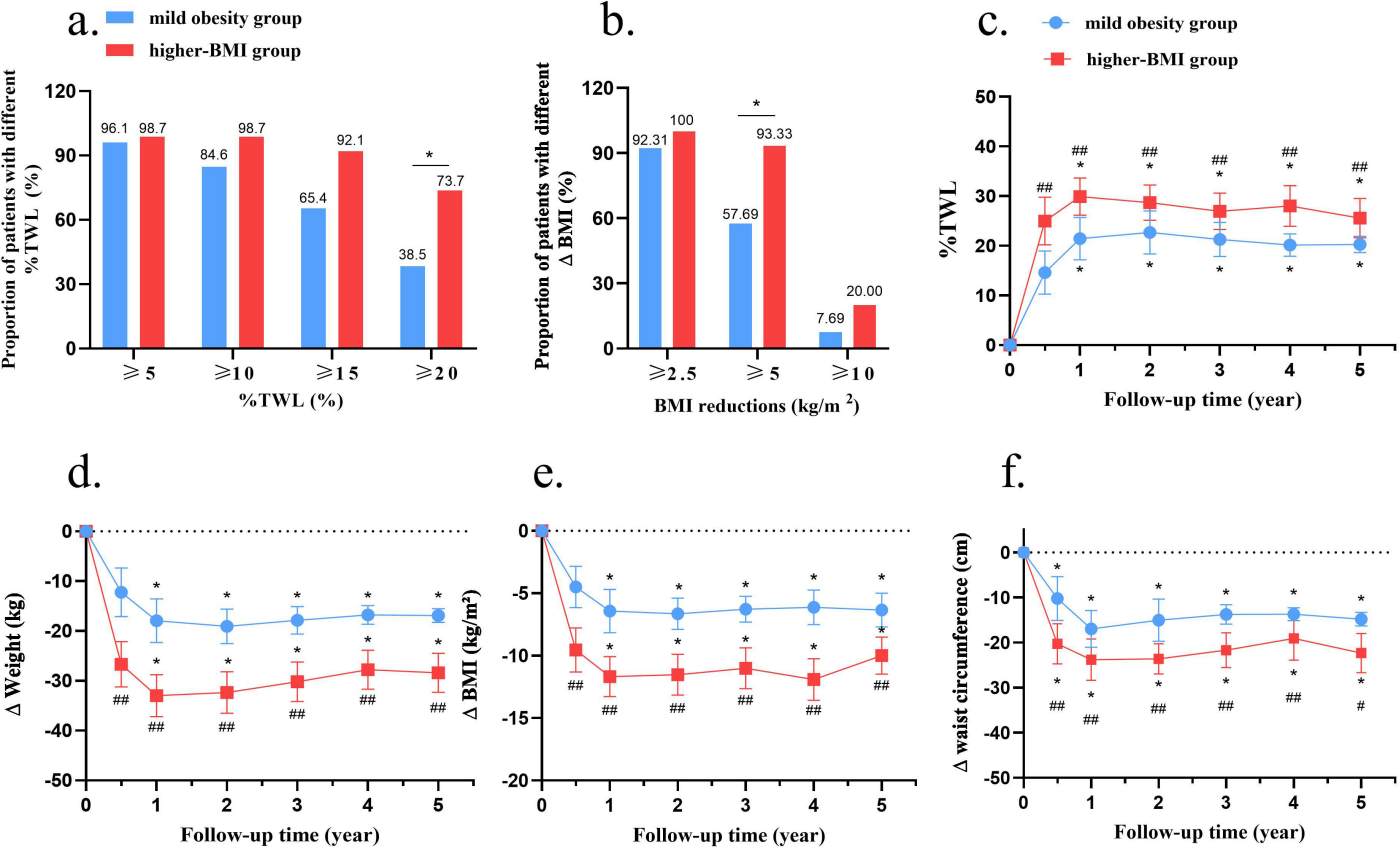
Comparison of weight-related outcomes between groups. **(a)** Proportions of patients achieving %TWL ≥TWL ≥TWLe ≥TWLe and ≥ndLe **(b)** Proportions of patients achieving ΔBMI ≥BMIev ≥BMI and ≥ndIe **(c)** Changes in %TWL at each follow-up visit; **(d)** Changes in weight at each follow-up visit; **(e)** Changes in BMI at each follow-up visit; **(f)** Changes in waist circumference at each follow-up visit. BMI, body mass index; TWL, total weight loss. *P < 0.05 for **(a, b)**; *P < 0.05 vs. baseline for **(c-f)**; #*P* < 0.05, ##*P* < 0.01 vs. mild obesity group at the same follow-up time.

At the final follow-up, both groups achieved substantial and durable weight reduction. Patients in the higher-BMI group showed a higher %TWL compared to those with mild obesity (27.3 ± 1.3% vs. 18.0 ± 1.7%), with an adjusted mean difference of -9.3% (95% CI, -14.0 to -4.7, *P* < 0.01) (**Table 1**). Both groups showed greater reductions in weight, BMI, waist circumference and waist-to-height ratio from baseline (all *P* < 0.01 vs baseline). A higher proportion of the higher-BMI group achieved > 20% TWL and a BMI decrease ≥ 5 kg/m² (*P* < 0.05; **Figures 4a, b**). In contrast, the proportions achieving ≥ 5%, ≥ 10%, or ≥ 15% TWL, or BMI reductions ≥ 2.5 or ≥ 10 kg/m², were similar between groups (**Figures 4a, b**). Notably, a greater proportion of patients in the mild obesity group reached a normal BMI (< 24.0 kg/m²) by the end of follow-up (43.2% vs 9.2%, *P* < 0.01), while the proportions remaining overweight (24.0–27.9 kg/m²) were comparable (**Table 1**).

Longitudinal weight-trajectory curves showed rapid weight loss within the first year after surgery, followed by long-term stabilization in both groups (**Figures 4c–f**). Throughout the follow-up period from 6 months onward, the higher-BMI group consistently demonstrated greater %TWL and more pronounced reductions in body weight, BMI, and waist circumference than the mild obesity group (*P* < 0.01–0.05; **Table 1**, **Figures 4c–f**).

### 3.4 Postoperative complications

Nutritional parameters at the final follow-up are summarized in **Table 1**. Although serum hemoglobin levels decreased in both groups, mean values remained within the normal range. Serum albumin, ferritin, folate, vitamin B12, and 25-hydroxyvitamin D levels remained stable relative to baseline, with no significant differences between groups.

The overall incidence of postoperative complications was low and comparable between groups (*P* > 0.05, **Table 2**). In the mild obesity group, 23 adverse events were recorded, including one gallstone requiring cholecystectomy, one case of hypoalbuminemia, eight cases of iron-deficiency anemia, ten cases of low ferritin without overt anemia, and two cases of bone demineralization (osteopenia or osteoporosis). In the higher-BMI group, 74 events were documented: infections (n = 3), postoperative hemorrhages (n = 4), anastomotic leaks (n = 2), gallstones (n = 6), hypoalbuminemia (n = 11), iron-deficiency anemia (n = 16), low ferritin without overt anemia (n = 28), and bone demineralization (n = 4).

**Table 2:** Postoperative complications.

Complications	Mild obesity group (*n* = 26)	Higher-BMI group (*n* = 76)	p
nfection (n, %)	0 (0%)	3 (3.9%)	0.14
Postoperative hemorrhage (n, %)	0 (0%)	4 (5.3%)	0.11
Anastomotic leak (n, %)	1 (3.8%)	2 (2.6%)	0.84
Gallstone (n, %)	1 (3.8%)	6 (7.9%)	0.37
Hypoalbuminemia (n, %)	1 (3.8%)	11 (14.5%)	0.15
Iron-deficiency anemia (n, %)	8 (30.8%)	16 (21.1%)	0.31
Low ferritin (n, %)	10 (38.5%)	28 (36.8%)	0.88
Bone loss (n, %)	2 (7.7%)	4 (5.3%)	0.89

All complications were effectively managed with standard interventions, including medical therapy, targeted nutritional supplementation, and endoscopic stenting where applicable. No mortality or long-term disability occurred.

## 4 Discussion

In this study, we evaluated mid-term (approximately 5-year) outcomes of MBS in Asian patients with mild obesity (BMI 27.5–32.5 kg/m²) compared with those with higher BMI. Although absolute weight loss was smaller in the mild obesity group, the proportion of patients achieving the composite metabolic-success endpoint was comparable between groups, indicating similar overall cardiometabolic benefit. These findings raise the possibility that BMI alone may not be the sole determinant of surgical eligibility in Asian patients with metabolic disease, particularly given the high metabolic burden observed at lower BMI levels in this population (4, 6, 23, 36).

The composite endpoint integrated glycemic control, lipid profile, blood pressure, and insulin resistance, providing a multidimensional assessment of cardiometabolic recovery (35). The comparable composite success observed despite differences in absolute weight loss indicates that metabolic benefit after surgery extends beyond simple weight reduction and reflects broader improvements in metabolic regulation (11, 12). This is especially relevant in Asian populations, where adverse metabolic profiles often emerge at relatively low BMI levels (21, 22, 36).

Both BMI groups demonstrated substantial remission or improvement in T2DM, hypertension, MAFLD, and hyperuricemia, consistent with previous long-term studies of metabolic surgery (11, 12, 37, 38). The greater remission of hypertension and more pronounced reduction in dyslipidemia in the higher-BMI group likely reflect their higher baseline disease burden rather than differential treatment responsiveness.

Why did patients with mild obesity see similar metabolic improvements even though they lost less weight? Several mechanisms might explain this. First, surgery improves blood sugar through pathways beyond just weight loss—such as boosting GLP-1 secretion, changing bile acid metabolism, and shifting gut microbiota (39, 40). Second, blood pressure may improve after surgery because of reduced sympathetic nervous system activity, better sodium handling, and lower angiotensin II sensitivity (41). Third, the drop in liver enzymes and uric acid suggests benefits for liver fat and purine metabolism that don’t seem to depend on how much weight was lost.

Importantly, these findings suggest that patients with mild obesity, despite achieving smaller absolute weight loss, can derive clinically meaningful cardiometabolic benefits from MBS. This observation supports the concept that metabolic improvement following surgery is not solely dependent on the degree of weight reduction but involves complex physiological adaptations that may be equally accessible to patients with lower baseline BMI (36).

Beyond individual comorbidities, our findings underscore the importance of metabolic phenotype over BMI alone. Factors such as visceral adiposity, insulin resistance, and underlying metabolic susceptibility may more strongly influence surgical outcomes than baseline body mass (14, 24). The marked improvement in insulin resistance observed in both groups likely represents an upstream mechanism contributing to global metabolic recovery. These observations support a metabolically oriented approach to surgical candidacy, particularly in Asian patients who develop metabolic complications at lower BMI levels (13, 36).

The present study adds important mid-term evidence for patients with BMI <30–35 kg/m². While previous Chinese studies in this range have largely reported short-term outcomes (17, 18), our data demonstrate sustained benefits over approximately 5 years. Improvements in glycemic control, lipid metabolism, blood pressure, and obesity-related comorbidities were maintained beyond 3 years, consistent with long-term randomized and observational evidence supporting durable metabolic benefit after surgery (11, 12, 15, 37).

Pharmacological therapy, particularly GLP-1 receptor agonists, provides substantial weight-loss and metabolic benefit but generally requires continuous treatment, with weight regain commonly observed after discontinuation (9, 10). In contrast, metabolic surgery yields more sustained cardiometabolic improvement (11, 12). Randomized trials, meta-analyses, and emerging Asian data indicate superior long-term cardiometabolic protection with surgical intervention compared with non-surgical strategies (20, 42, 43). Importantly, accumulating evidence suggests that meaningful benefit can also be achieved in patients with lower BMI (20, 44).

A clinically meaningful finding was that more patients with mild obesity achieved normal weight (BMI <24 kg/m²) after surgery. Reaching a healthy BMI may provide metabolic advantages beyond weight loss alone. Previous studies indicate that patients who normalize their weight after surgery experience greater metabolic improvements than those who remain overweight or obese (44). This suggests weight normalization is an important target and could support earlier intervention in selected patients with mild obesity. However, our findings are hypothesis-generating. The comparable composite success rate comes from a small sample and may reflect type II error or baseline imbalances rather than true equivalence.

The safety profile was comparable between groups, with low complication rates consistent with contemporary metabolic-surgery standards (33). These findings align with current guideline recommendations and registry data demonstrating that metabolic surgery can be safely performed in appropriately selected lower-BMI patients within experienced multidisciplinary centers (13, 33).

Several limitations should be acknowledged. Although ANCOVA was used to adjust for measured baseline covariates, residual confounding by unmeasured variables (e.g., dietary habits, physical activity, medication adherence) cannot be fully excluded. The relatively small sample size, particularly in the mild obesity group, limits the statistical power to detect clinically meaningful differences. Furthermore, we could not perform a separate analysis of the most debated subgroup—patients with BMI 27.5–30 kg/m² without T2DM or advanced metabolic disease—because fewer than five patients in our mild obesity group met these criteria. Our findings should not be extrapolated to this lower-risk population. The retrospective single-center design and non-randomized procedure selection may also limit the study’s generalizability. Certain long-term endpoints, including cardiovascular events, microvascular complications, and quality of life, were not assessed. The absence of a non-surgical control group precludes direct comparison with optimized medical therapy. Future multicenter prospective studies and registry-based analyses are warranted to evaluate long-term cardiometabolic outcomes, compare surgical versus advanced medical therapies, including multi-target GLP-1 based agents, and refine selection criteria for patients with BMI <32.5 kg/m² (19, 20). Additionally, the intensive, multidisciplinary postoperative follow-up provided at our high-volume center, including regular visits with metabolic surgeons, endocrinologists, dietitians, and specialized nurses, may have contributed to the favorable outcomes and low complication rates observed in both groups. This close monitoring facilitated early detection and management of nutritional deficiencies and other complications, which may improve clinical outcomes compared with real-world settings where follow-up is less structured. Therefore, our findings may have limited generalizability to centers with less intensive follow-up protocols.

In this cohort of Asian patients with BMI 27.5–32.5 kg/m², MBS was safe and provided durable metabolic benefits over approximately 5 years of follow-up. After adjusting for baseline differences, those with mild obesity achieved similar improvements in insulin resistance, blood pressure, liver function, and lipids, despite losing less weight. However, the small sample size, lack of a non-surgical control group, and the hypothesis-generating nature of these findings mean that larger studies are needed before we can draw definitive conclusions about surgical indications. Future investigations should focus on large, multicenter studies with sufficient statistical power to evaluate patients with BMI 27.5–30 kg/m² who do not have advanced metabolic comorbidities. Well-designed randomized trials comparing surgery with current pharmacologic therapies—including GLP-1 receptor agonists and multi-target agents—are also warranted. Finally, long-term studies tracking hard endpoints such as major adverse cardiovascular events, microvascular complications, and health-related quality of life will help strengthen the evidence base for surgical intervention in this population.

## Data Availability

The raw data supporting the conclusions of this article will be made available by the authors, without undue reservation.
